# Diagnosis and treatment of patients with antiphospholipid syndrome: a mixed-method evaluation of care in The Netherlands

**DOI:** 10.1093/rap/rkaa021

**Published:** 2020-06-12

**Authors:** Mirthe J Klein Haneveld, Caro H C Lemmen, Tammo E Brunekreef, Marc Bijl, A J Gerard Jansen, Karina de Leeuw, Julia Spierings, Maarten Limper, ARCH Study Group, Titia Lely, Renate van der Molen, Rolf Urbanus, Nyika Kruyt, Marcel van de Ree, Judith Potjewijd, Gerie Brandts, Jamy Scheerhoorn-Pullen, Carolijn de Bresser, Sander Otter, Julia Berentschot, Nicole Hulsebosch, Rita Schriemer

**Affiliations:** r1 Department of Rheumatology and Clinical Immunology, University Medical Centre Utrecht, Utrecht; r2 Department of Internal Medicine and Rheumatology, Martini Hospital, Groningen; r3 Department of Haematology, ErasmusMC, University Medical Centre Rotterdam, Rotterdam; r4 Department of Rheumatology and Clinical Immunology, University Medical Centre Groningen, Groningen, The Netherlands

**Keywords:** antiphospholipid syndrome, quality of care, qualitative research, medical records, unmet patient needs

## Abstract

**Objectives:**

The aims were to gain insight into the care provided to patients with APS in The Netherlands and to identify areas for improvement from the perspective of both patients and medical specialists.

**Methods:**

APS care was evaluated using qualitative and quantitative methods. Perspectives on APS care were explored using semi-structured interviews with medical specialists, patient focus groups and a cross-sectional, online patient survey. In order to assess current practice, medical records were reviewed retrospectively to collect data on clinical and laboratory manifestations and pharmacological treatment in six Dutch hospitals.

**Results:**

Fourteen medical specialists were interviewed, 14 patients participated in the focus groups and 79 patients completed the survey. Medical records of 237 patients were reviewed. Medical record review showed that only one-third of patients were diagnosed with APS within 3 months after entering specialist care. The diagnostic approach and management varied between centres and specialists. Almost 10% of all patients and 7% of triple-positive patients with thrombotic APS were not receiving any anticoagulant treatment at the time of medical record review. Correspondingly, poor recognition and fragmentation of care were reported as the main problems by medical specialists. Additionally, patients reported the lack of accessible, reliable patient education, psychosocial support and trust in physicians as important points for improvement.

**Conclusion:**

Delayed diagnosis, variability in management strategies and fragmentation of care were important limitations of APS care identified in this study. A remarkable 10% of patients did not receive any anticoagulant treatment.

**Key messages**

- Delayed diagnosis is common among patients with APS.
- APS treatment strategies vary between medical specialists and hospitals.
- Unmet APS patient needs include patient education, psychosocial support and trust in physicians.

## Introduction

The APS is a rare autoimmune disease affecting ∼1000–2000 patients in The Netherlands [1, 2]. A diagnosis of APS is generally made when a patient meets the classification criteria: vascular thrombosis and/or pregnancy morbidity, such as repeated spontaneous abortion, unexplained fetal death and preterm birth, in the repeated presence of circulating aPL targeted at aCL and/or β2-glycoprotein I (β2GPI) and/or lupus anticoagulant (LAC) with an interval of ≥12 weeks [1, 2]. APS is associated with a variety of non-criteria clinical manifestations, such as thrombocytopenia, renal microangiopathy, heart valve disease, livedo reticularis and migraine [1, 2]. It often occurs in isolation, but can be found in association with SLE and other autoimmune diseases [1, 2]. Lifelong anticoagulation is the mainstay of therapy for thrombotic APS owing to the high risk of relapse; for obstetrical APS, treatment exists of low-dose aspirin and prophylactic low-molecular-weight heparin during pregnancy [2, 3]. Additionally, immunomodulatory drugs, such as HCQ, are recommended for secondary APS in SLE patients [2, 3]. Optimal care for APS is challenging because of its rare occurrence, variation in diagnostic test assays and interpretation, heterogeneous clinical manifestations and subsequent multidisciplinary character. A review of clinical practice guidelines concluded that a formal guideline covering all relevant aspects of APS diagnosis and treatment is missing [4]. Given that large randomized controlled trials for treatment of APS are rarely performed, the development of evidence-based guidelines, such as the 2019 EULAR recommendations for the management of APS, remains very challenging [3, 4]. As a consequence, variation in treatment strategies between medical specialists and centres is, presumably, high [2–4].

Few studies have investigated the experiences regarding APS care of patients and physicians. A questionnaire distributed among patients in the UK pointed out that there was a long delay between first symptoms and diagnosis, with a median duration of 3 years, in addition to a lack of awareness of APS among general practitioners and medical specialists [5]. Qualitative studies into experiences of APS patients described the impact of living in uncertainty and delayed diagnosis [6–8]. Patient representatives highlighted the need for improved patient education and monitoring [4]. However, these unmet patient needs require more research attention [4–8].

The Dutch Arthritis Research and Collaboration Hub (ARCH) aims to improve care for rare autoimmune diseases, including APS [2]. Using both qualitative and quantitative methods, the aims of the present study were to gain insight into the care currently provided to patients with APS in The Netherlands and to identify unmet needs and areas for improvement from the perspectives of patients and medical specialists from different centres and disciplines.

## Methods

### Design

This study adopted a mixed-method design to collect qualitative and quantitative data from the perspectives of patients and medical specialists. We collected data in three stages. Firstly, qualitative data were collected from focus group sessions with patients and interviews with medical specialists. Secondly, an online survey was distributed among patients. Thirdly, medical records in university and general hospitals were reviewed to evaluate variation in patient characteristics, the diagnostic process and management between centres. Ethical approval was given by the Medical Ethical Committee of the University Medical Centre Utrecht (METC number 18-508) and all participating hospitals.

### Setting and participants

Twenty medical specialists known to have a special interest in thrombotic conditions were invited to participate in individual interviews between June and September 2018. Medical specialists were selected by three ARCH APS working group members in order to reach a heterogeneous sample with regard to sex, discipline (neurology, haematology, clinical immunology, rheumatology, gynaecology and vascular medicine), patient load and type of hospital in The Netherlands. All interviews with medical specialists were held by telephone by the same researcher (J.S.) and lasted between 30 and 75 min. The researcher (female, rheumatologist, researcher) was not affiliated with the participants.

Patients with APS were recruited to participate in focus groups by the national patient organization [*Nationale Vereniging voor Lupus, APS, Sclerodermie en MCTD* (NVLE)]. Four focus group sessions, with three or four participants per group, took place between June and November 2018 at a meeting point centrally located in the country. Before the focus group, participants were asked to fill out a questionnaire on sociodemographic information and disease characteristics. All focus groups were moderated by one researcher (J.S.) in the presence of a representative of the patient organization and lasted between 120 and 150 min.

Medical specialists from four university hospitals [University Medical Centre Utrecht (UMCU), Erasmus Medical Centre Rotterdam (EMC), Universal Medical Centre Groningen (UMCG) and Maastricht University Medical Centre (MUMC)] and three general hospitals [Diakonessenhuis Utrecht, Hospital Group Twente (ZGT) Almelo/Hengelo and Martini Hospital Groningen] invited a total of 109 patients by mail to participate in the online self-administered survey. The inclusion criterion was a clinical diagnosis of APS according to their medical specialist. Patients could sign up for participation by sending an email to the researcher with their consent. Subsequently, they received a link to the survey in Castor Electronic Data Capture (EDC) [9]. The survey was open from November 2018 until June 2019.

Between March and May 2019, medical records from four university hospitals (UMCU, EMC, UMCG and MUMC) and two general hospitals (Diakonessenhuis Utrecht and ZGT) were reviewed. Each centre compiled a list of patients for whom aPL measurement was requested at the laboratory. Patients were randomly selected from this list and included in the study if the following inclusion criteria were met: a clinical diagnosis of APS according to the coordinating physician; and availability of data regarding APS in the patient record. Inclusion of patients continued until a maximum of 50 patients was reached or no new APS patients could be identified.

### Data collection

Two rheumatologists and a clinical immunologist formulated an interview guide for semi-structured interviews. It was tested by an advisory group consisting of patients and medical specialists of the ARCH APS working group (Supplementary Material, section Interview Guide, available at *Rheumatology Advances in Practice* online) A semi-structured approach was chosen because it ensured that all topics were addressed but left room for flexibility in pursuing participants’ interests and expertise [10]. The focus groups had a similar semi-structured approach. The diagnostic process, management after diagnosis, provision of information and psychosocial support were addressed. Participants were asked to share the main challenges and unmet needs and to suggest relevant process and outcome measures that should be used as quality indicators for APS care. The interviews and focus group sessions were recorded and transcribed verbatim by three independent researchers (M.J.K.H., C.H.C.L. and 'J.S.).

Data collection for the online survey and medical record review was done in Castor EDC [9]. The online survey was composed and tested in a similar manner to the interview guide (Supplementary Material, section Online Survey, available at *Rheumatology Advances in Practice* online). Data for medical record review were collected using a case report form. The relevant process and outcome measures mentioned in focus groups and interviews were incorporated into the case report form (Supplementary Table S1, available at *Rheumatology Advances in Practice* online). The data collected included demographic information, duration of illness, time until diagnosis, clinical and laboratory criteria of APS, non-criteria disease manifestations, medication use, complications of treatment and information regarding disease management.

### Data analysis

Two independently working researchers (M.J.K.H. and C.H.C.L.) analysed the data from interviews and focus group sessions mostly in a deductive fashion [11]. The researchers first familiarized themselves with the data by thoroughly reading the transcripts and writing down initial ideas. Themes were identified using the interview guide. Additionally, topics that were frequently brought up by participants were considered as themes or subthemes. Subsequently, the two researchers discussed the identified themes and assessed their internal homogeneity and external heterogeneity. The results were summarized by the two researchers, discussed in the ARCH APS working group and used to compose the questions for the survey. The consolidated criteria for reporting qualitative research (COREQ) are reported in Supplementary Table S2, available at *Rheumatology Advances in Practice* online [12].

Quantitative data were processed using IBM SPSS Statistics for Windows, v.25.0.

## Results

### Specialist interviews, patient focus groups and patient survey

#### Characteristics of study participants

Of the 20 invited medical specialists, four did not respond and two did not want to participate owing to time limitations. The characteristics of 14 interviewed medical specialists are displayed in Table 1. For the online survey, 109 patients were invited; the response rate was 72.5%. Demographic and clinical characteristics and the experiences of 14 focus group participants and 79 patients who filled out the digital survey are described in Table 2.

**Table 1:** Demographic characteristics of interviewed specialists (*n* = 14)

Characteristic	Value	
Specialty, *n* (%)		
Clinical immunologist	3	(21.4)
Internist in vascular medicine	3	(21.4)
Rheumatologist	3	(21.4)
Neurologist	2	(14.2)
Haematologist	2	(14.2)
Gynaecologist	1	(1.2)
Age median (range), years (*n* =12)	44	(37–58)
Hospital type, *n* (%)		
University hospital	9	(64.3)
General tof patients per year, *n* (%)
<5	3	(21.4)
5–10	2	(14.2)
10–30	3	(21.4)
>30	6	(42.9)
Sex, *n* (%)
Male	10	(71.4)
Female	4	(28.6)

**Table 2:** Characteristics and experiences of focus group participants (*n* = 14) and survey respondents (*n* = 79)

Demographic and clinical characteristics		Focus group participants (*n* = 14)		Survey respondents (*n* = 79)	
Age, median (range)		46 (27–65)		53 (26–77)	
Sex, *n* (%)	Female	13	(92.9)	68	(86.1)
	Male	1	(7.1)	11	(13.9)
Highest completed education, *n* (%)	Secondary vocational training or less	4	(28.6)	45	(57.0)
	Higher professional or university education^a^	10	(71.4)	34	(43.0)
Treatment centre, *n* (%)	University hospital	7	(50.0)	67	(84.8)
	General hospital	5	(35.7)	6	(7.6)
	Other/do not know^b^	2	(14.3)	6	(7.6)
Duration of disease, median (range), years		6.5 (0–22)		7 (1–27)	
Duration of symptoms before diagnosis, *n* (%)	>5 years	3	(21.4%)	29	(36.7)
	3–5 years	0	(0.0)	4	(5.1)
	2–3 years	3	(21.4)	3	(3.8)
	1 year	0	(0.0)	5	(6.3)
	6 months	1	(7.1)	8	(10.1)
	<6 months	4	(28.6)	11	(13.9)
	Do not know	3	(21.4)	19	(24.1)
Other rheumatological disease, *n* (%)	No, primary APS	7	(50.0)	33	(41.7)
	SLE	6	(42.8)	21	(26.6)
	Other/do not know^c^	1	(14.2)	25	(31.6)
Manifestations of disease, *n* (%)	Deep venous thrombosis	3	(21.4)	37	(46.8)
	Cerebrovascular accident	5	(35.7)	23	(29.1)
	Transient ischaemic attack	3	(21.4)	18	(22.7)
	Obstetric manifestation	3	(21.4)	30	(38.0)
	Thrombocytopenia	1	(7.1)	11	(13.9)
	Livedo reticularis	5	(35.7)	10	(12.7)
	Endocarditis	1	(7.1)	5	(6.3)
	Migraine	6	(42.8)	12	(15.2)
	Other	7	(50.0)	18	(22.8)
Experienced limitation owing to APS, *n* (%)	Work	6	(42.8)	40	(50.6)
	Travel	8	(57.1)	34	(43.0)
	Daily functioning	6	(42.8)	43	(54.4)

a Higher professional or university education is defined as Dutch higher vocational training (HBO) or university level. Secondary vocational training or less is defined as Dutch secondary vocational training (MBO), secondary education (VWO, HAVO, VMBO) or primary education. ^b^Not under treatment at any medical hospital or shared care between multiple hospitals. ^c^Among others: SS, RA.

#### Perspectives on the diagnostic process

The importance of being taken seriously by medical specialists and general practitioners and the necessity of referral to expert centres were recurring themes in the focus groups and survey responses. Several patients felt that they were ‘being fobbed off’ by physicians; two focus group participants remarked that their health problems had been interpreted initially as psychosomatic. Self-reported time to diagnosis in focus groups and survey responses varied widely, ranging from <1 month to >5 years. Overall, the time to diagnosis had a strong negative impact on satisfaction with the diagnostic process among the focus group participants. However, only 7.6 and 8.9% of survey respondents considered delayed diagnosis and, respectively, insufficient recognition of APS by physicians to be the main obstacles in APS care.

According to medical specialists, the main challenges in the diagnostic process are insufficient recognition of APS by medical specialists (10/14) and general practitioners (7/14) in addition to the absence of evidence-based diagnostic guidelines (3/14). One specialist considered the quality of laboratory diagnostics to be a main challenge. Four medical specialists remarked that the diagnostic process of APS requires more expertise compared with the management of APS after diagnosis.

#### Perspectives on management after diagnosis

Patients felt the need to orchestrate their own care, because they experienced a lack of collaboration and communication between medical specialists and between medical specialists and medical services, i.e. the anticoagulation service (Dutch: Trombosedienst). This made them as patients responsible for an adequate exchange of information about their disease and medication use, which could be particularly worrisome for patients when they were critically ill and required emergency care. Several participants expressed the need for a document explaining their condition to show at emergency departments. Correspondingly, limitations in the exchange of patient information between hospitals were mentioned as a burden by 50% of the interviewed medical specialists.

Another main challenge in APS care reported by 8 of 14 medical specialists was the lack of evidence-based treatment guidelines. The absence of uniform guidelines was thought to contribute to variation in treatment strategies and to have a negative impact on the quality of care. Patients recognized this issue and referred to uncertainty regarding the management of anticoagulation in the context of surgical procedures.

Ten medical specialists agreed that multidisciplinary consultation should be possible for all patients; however, only six reported that this was available at their hospital. Twelve medical specialists agreed to the statement that APS care is fragmented; eight agreed that improved communication is necessary to improve cooperation between physicians.

#### Perspectives on information provision, psychosocial support and daily functioning

Patient education was considered to be insufficient by 8 of 14 interviewed medical specialists, 11 of 14 focus group participants and 41.8% of survey respondents. Patients identified this as a big challenge in APS care and a good measure for quality of care. Patients particularly needed information about the wide range of symptoms attributed to APS, the impact of APS on daily life and prognostic information. A key provider of patient information and support is the patient organization for APS, although 3 of 14 medical specialists and 38.0% of survey respondents were unfamiliar with this organization. Some patients and medical specialists proposed that specialized nurses might assist in patient education.

A minority of focus group participants and survey respondents (16.5%) was offered psychological assistance after diagnosis, although more than half of respondents would have welcomed this support. According to interviewed medical specialists, a lack of time, not being the coordinating physician and no apparent need for psychosocial assistance were barriers to the provision of psychosocial support.

The last unmet need identified in APS care was the need for support in coping with limitations in daily functioning (reported by 54.4% of survey respondents) and support with occupational hurdles. Focus group participants therefore included employment status and satisfactory daily functioning as important outcomes to measure in APS care.

**Fig. 1:**
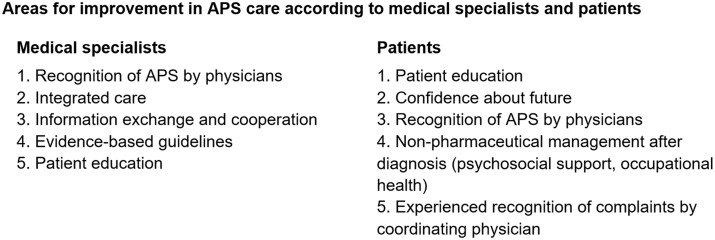
Areas for improvement in APS care according to medical specialists and patients resulting from survey

Fig. 1 summarizes the relevant areas for improvement as reported by medical specialists and survey respondents. Participant quotations are provided in Supplementary Table S3, available at *Rheumatology Advances in Practice* online.

### Medical record review

#### Clinical and laboratory criteria and diagnostic process

Medical records of 237 patients were reviewed. Demographic, clinical and laboratory characteristics are displayed in Table 3. Of all patients, 70.9% had thrombosis, 40.2% experienced obstetric complication(s) and 22.4% experienced both. In 9.3% of patients there were no thrombotic or obstetric events, but a diagnosis of APS was made because of non-criteria manifestations. The median number of recorded non-criteria manifestations was 1.0 (interquartile range 0.0–2.0, range 0.0–5.0). The most common non-criteria manifestations were thrombocytopenia (25.3%), pre-eclampsia/haemolysis, elevated liver enzymes and low platelets syndrome (13.4% of female patients), livedo reticularis (12.7%), migraine (11.8%) and valvular heart disease (10.1%).

**Table 3:** Demographic, clinical and laboratory characteristics of patients included in medical record review

Patient characteristics		University hospitals (n = 192)		General hospitals (n = 45)		All patients (n = 237)	
Age, mean, s.d., years		46	13	49	15	47	13
Duration of disease, median, IQR, years (*n* =235)		6	2–14	3	2–8	5	2–14
Sex, %	Male	15.1		31.1		18.1	
	Female	84.9		68.9		81.9	
Type of APS, %	Primary APS	61.5		86.7		66.2	
	Secondary APS	38.5		13.3		33.8	
	Of which SLE (*n* =52)	76.5		80.0		76.8	
Treatment centre, %	University hospital					81.0	
	General hospital					19.0	
Deceased, %		2.1		2.2		2.1	

Time until diagnosis		Median	IQR	*n*
Duration until under specialist treatment, months	All patients	0.0	0.0–3.0	212
	University hospital	0.0	0.0–3.0	171
	General hospital	0.0	0.0–2.0	41
Duration under specialist treatment until diagnosis, months	All patients	6.0	2.0–24.0	218
	University hospital	7.0	2.0–24.0	175
	General hospital	4.0	1.0–12.0	43
Diagnosed with APS within 3 months after entering specialist care, %, *n*	All patients	34.9		218
	University hospital	32.6		175
	General hospital	44.2		43

Clinical and laboratory criteria			University hospital (*n*=192)	General hospital (*n* =45)	All patients (*n*= 237)
Criteria clinical manifestations	Vascular thrombosis, %		72.9	62.2	70.9
	Number of events, median (IQR) (*n* = 168)		2.0 (1.0–3.0)	1.0 (1.0–2.0)	2.0 (1.0–3.0)
	Obstetric complication(s)		42.2	17.8	40.2
	Number of events, median (IQR) (*n* =100)		1.0 (1.0–2.0)	2.0 (1.0–3.0)	1.0 (1.0–3.0)
	Both thrombosis and obstetric complication(s), %		25.5	0.0	22.4
	Neither thrombosis nor obstetric complication(s), %		6.8	20.0	9.3
Non-criteria clinical manifestations	Thrombocytopenia, %		26.0	22.2	25.3
	Livedo reticularis, %		14.1	6.7	12.7
	Migraine, %		12.0	11.1	11.8
	Pre-eclampsia/HELLP syndrome, %; % females		13.0; 15.3	2.2; 3.2	11.0; 13.4
	Valvular heart disease, %		10.9	6.7	10.1
	Cutaneous ulceration, %		8.9	2.2	7.6
	Insult, %		7.8	2.2	6.8
	aPL-related nephropathy, %		4.7	4.4	4.6
	Chorea, %		3.1	0	2.5
	Superficial venous thrombosis, %		2.6	0	2.1
	Intra-uterine growth retardation, %; % females		2.6; 3.1	0	2.1; 2.6
Laboratory criteria	LAC	Prevalence, %	53.1	44.4	51.5
		Assessed in ≥2 samples, % (*n* = 122)	78.4	85.0	79.5
	aCL	Prevalence, %	79.2	57.8	75.1
		Assessed in ≥2 samples, % (*n* = 178)	89.5	84.6	88.8
	Anti-β2GP	Prevalence, %	47.9	48.9	48.1
		Assessed in ≥2 samples, % (*n* = 114)	85.8	72.7	82.5
	Seronegative for all 3 aPL, %		7.8	6.7	7.6
	Triple positive, %		25.0	17.8	23.6
Total number of positive criteria	Total number of elevated aPL, median (IQR)		2.0 (2.0–2.5)	1.0 (1.0–2.0)	2.0 (1.0–2.0)
	Total number of positive clinical and laboratory criteria, median (IQR)		3.0 (2.0–4.0)	2.0 (2.0–3.0)	3.0 (2.0–4.0)

β2GPI: anti-β2-glycoprotein I antibodies; HELLP: haemolysis, elevated liver enzymes and low platelets; IQR: interquartile range; LAC: lupus anticoagulant.

aPL were elevated in 92.4% of patients: aCL, LAC and anti-β2GPI antibodies were detected in 75.1, 51.9 and 48.1%, respectively. In 7.6% of patients, seronegativity for all measured aPL was described. Fifty-five patients (23.6%) were ‘triple positive’, of whom 42 experienced thrombotic manifestations and 13 had obstetric APS. In 84.3% of laboratory measurements, aPL status was assessed in at least two separate samples.

The median duration between the first recorded disease manifestation and receiving specialist care was 0 months. The median time until diagnosis after entering specialist care was 6 months (interquartile range 2–24 months). In 32.1% of patients, the diagnosis of APS was established within 3 months.

#### Management after diagnosis

In 81.8% of patients, the coordinating physician could be identified from medical case records; this was most often a rheumatologist/internist clinical immunologist (39.7%) or general internist (28.7%). In 18.2% of patients, the coordinating physician could not be identified from the medical record or patients were not under regular follow-up with any medical specialist. Gynaecologists, neurologists and haematologists were frequently involved in the diagnostic process and management (in 36.7, 34.6 and 23.2%, respectively), but were less often the coordinating physician (in 4.2, 0.4 and 4.2%, respectively). The number of medical disciplines involved in diagnosis and management was one in 19.4%, two in 38.4%, three in 21.9% and four or more in 20.3% of patients. In 45.6% of case, patients were discussed in a multidisciplinary consultation meeting. A specialized nurse was involved with 14.8% of patients.

**Fig. 2:**
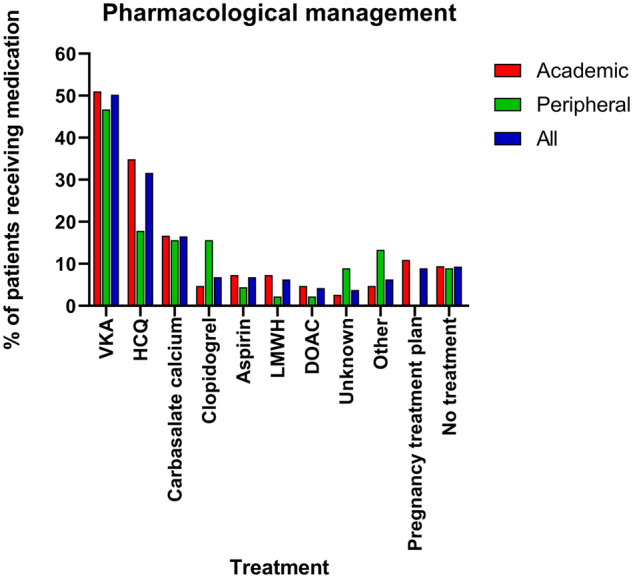
Pharmacological management of patients included in medical record review Patients who received multiple medication types for management of APS count towards all received mentioned medications. DOAC: direct oral anticoagulant; LMWH: low-molecular-weight heparin; Other: amongst others, plasmapheresis, IVIG; Pregnancy treatment plan: documentation of plan to start treatment in case of pregnancy; VKA: vitamin K antagonist.

In Fig. 2, pharmacological management is displayed. Most patients were treated using vitamin K antagonists, HCQ and/or carbasalate calcium/ascal. No anticoagulant treatment was provided in 9.3% of all patients. This was the case in 5 of 42 triple positive patients, of whom 3 had thrombotic APS (7.1%) with an indication for lifelong anticoagulation.

In 26.2% of patients, a next thrombotic or obstetric event occurred after diagnosis. Complications of treatment occurred in 25.4%: bleeding and intolerance of medication were reported in 20.3 and 12.5%, respectively. End-organ damage, including permanent ischaemic events, neurological damage, amputation, catastrophic APS and heart or renal failure, was described in 24.5%.

Fitness for work was often not reported in medical records (47.2%). Of all patients, 28.7, 11.4 and 12.7% were demonstrated to be fully, partly fit or unfit for work, respectively.

## Discussion

**Fig. 3:**
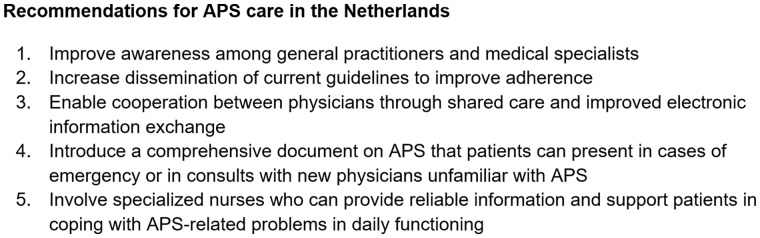
Recommendations for APS care in The Netherlands

In this mixed-method study, we evaluated current health care for patients with APS in The Netherlands. Delayed diagnosis, variation in management strategies and unmet needs with regard to patient education and self-management among APS patients were observed. Recommendations for improving APS care are provided in Fig. 3.

Both patients and medical specialists identified poor recognition of APS and, consequently, delayed diagnosis as a major obstruction to quality of care. The self-reported duration between the onset of any symptoms and diagnosis exceeded 5 years in more than one-third of patients. Moreover, only one-third of patients included in medical record review were diagnosed with APS within 3 months after entering specialist care. These findings are in line with previous research describing a long diagnostic delay in APS, during which patients experience uncertainty about their health [5, 6]. Our study found a median delay of 0 months between the first recorded disease manifestation and receiving specialist care; we hypothesize that this is because the event leading to specialist referral is often registered as the first disease manifestation. A cross-sectional Mexican study including 176 APS patients found that in patients who experienced both thrombosis and a non-criteria manifestation, non-criteria manifestations pre-dated the first thrombotic event in 28.7% [13]. Non-criteria manifestations, such as thrombocytopenia, pre-eclampsia/haemolysis, elevated liver enzymes and low platelets syndrome, livedo reticularis, migraine and valvular heart disease, all occurred in >10% of patients included in our study and might still be under-reported; these manifestations might thus both contribute to the diagnosis of APS and significantly influence the clinical condition of patients with APS.

Another remarkable finding was the variability in management strategies. Although recent consensus papers aim to support physicians in daily clinical decision-making, low quality and uptake of recommendations posed a major challenge according to interviewed medical specialists [2–4]. In our study, a considerable percentage of patients was not treated according to the latest insights. In 9.3% of patients, no anticoagulant treatment or treatment plan in case of pregnancy was provided at the time of medical record review; in particular, 7.1% of triple positive patients with thrombotic APS, bearing the highest risk of recurrent thrombosis, did not receive any anticoagulant treatment [2, 3]. Moreover, 4.2% of patients were treated with direct oral anticoagulants, which might be associated with a higher risk of recurrent thrombosis in APS [14]. In our study, the reasons underlying treatment decisions were not derived from medical records. Given that no bleeding complications were recorded in patients who did not receive anticoagulant treatment, previous adverse events are an unlikely reason for not prescribing medication. We hypothesize that unawareness among physicians might play a role in the variation in treatment. Furthermore, fragmentation of care results in challenges in communication between medical specialists within and between hospitals. Fragmentation of care is a well-documented problem in other rare and systemic diseases [15].

Lastly, we identified unmet patient needs in current health care. The most reported need was patient education about the disease and self-management support with regard to daily activities, work and psychological wellbeing. This need is reaffirmed by previous studies describing the impact of the disease on daily life in APS patients and, specifically, how the lack of knowledge about the disease results in uncertainty and stress [7, 8]. Previous research in other rare diseases likewise highlights patient education and provision of non-pharmacological care, such as psychological support, as a key need [4, 15–17]. Although several patients and medical specialists proposed that specialized nurses might play a role in providing reliable information and self-management support, nurses were involved with only a small proportion of patients.

A second important unmet need of patients was trust in physicians in general, because patients experienced that some medical specialists and general practitioners were unfamiliar with APS. Patients therefore felt the need to orchestrate their own care, such as taking an active role in organizing exchange of medical documentation between medical specialists, demanding diagnostic tests and proposing management strategies. This type of patient-directed interaction has been described previously as a widely experienced communication pattern among patients with rare diseases [18]. Remarkably, 10 of 14 focus group participants have received higher education; given that more than one-third of the Dutch population has limited health literacy, this level of control over disease management might not be achievable for all patients, potentially resulting in decreased quality of care [19]. Initiatives were proposed to bridge knowledge gaps, such as the provision of a document that explains APS and that can be used in emergency situations.

Our study has some limitations. Firstly, there could be selection bias, because a large proportion of included patients completed higher education and was treated at university hospitals. Secondly, only patients who were either familiar with the patient organization and able to travel or able use electronic communication methods could participate in the focus groups and survey, respectively. Therefore, the results might not be generalizable to all patients with APS. Thirdly, only medical specialists known to have a special interest in thrombotic conditions were interviewed. Lastly, we had to deal with missing or limited data and potential under-reporting in medical records. The strength of our study, however, is that, uniquely, we combined qualitative and quantitative research methods to evaluate care. To our knowledge, this is the first study of its kind in the field of APS. By integrating the perspectives of patients and medical specialists and medical record data from university and general hospitals across the country, it provides a comprehensive overview of current APS care in The Netherlands.

In conclusion, the main challenges in APS care in The Netherlands include delayed diagnosis, low quality and uptake of evidence-based recommendations, fragmentation of care and a burden placed on patients to orchestrate their own care. Unmet patient needs include patient education, support in daily functioning and trust in physicians. Despite the high risk of recurrent thrombosis, 7.1% of triple positive patients with thrombotic APS did not receive any anticoagulant treatment. Probable underlying factors for these challenges include the rare occurrence and heterogeneous character of APS. Future research should evaluate the clinical decision-making process in APS care and continue to address unmet patient needs. National and multidisciplinary collaboration and continuing education of physicians are required to improve APS care.

## Supplementary Material

rkaa027_Supplementary_Data
