# Erratum to: Diagnosis and treatment of patients with antiphospholipid syndrome: a mixed-method evaluation of care in The Netherlands

**DOI:** 10.1093/rap/rkab028

**Published:** 2021-06-21

**Authors:** Mirthe J Klein Haneveld, Caro H C Lemmen, Tammo E Brunekreef, Marc Bijl, A J Gerard Jansen, Karina de Leeuw, Julia Spierings, Maarten Limper, for the ARCH Study Group

**Affiliations:** 1 Department of Rheumatology and Clinical Immunology, University Medical Centre Utrecht, Utrecht; 2 Department of Internal Medicine and Rheumatology, Martini Hospital, Groningen; 3 Department of Haematology, ErasmusMC, University Medical Centre Rotterdam, Rotterdam; 4 Department of Rheumatology and Clinical Immunology, University Medical Centre Groningen, Groningen, The Netherlands Correspondence to: Maarten Limper, Department of Rheumatology and Clinical Immunology, Heidelberglaan 100, 3584 CX Utrecht, The Netherlands. E-mail: m.limper-2@umcutrecht.nl

Rheumatology Advances in Practice 2020. doi:10.1093/rap/rkaa021

"This paper originally erroneously published with the supplementary data from a different paper. This has now been corrected online."

## Supplementary Material

rkab028_Supplementary_Data

