# Supplementary material for: Diagnosis and treatment of patients with antiphospholipid syndrome: a mixed-method evaluation of care in The Netherlands
Source: Rheumatol Adv Pract. 2020 Jun 12;4(2):rkaa021. doi: 10.1093/rap/rkaa021 (PMC7474856; doi:10.1093/rap/rkaa021)
Supplement: rkaa027_Supplementary_Data [file rkaa027_supplementary_data.docx]

**SUPPLEMENTARY MATERIAL**

**Supplementary table S1 Classification of antidiabetic drugs**

| **Antidiabetic drug therapy** | **Medication** |
| --- | --- |
| Bolus Insulin | Insulin human regular  Insulin aspart  Insulin glulisine  Insulin lispro  Insulin aspart/Insulin aspart protamine  Insulin lispro/Insulin lispro protamine |
| Basal Insulin | Insulin human isophane (NPH)  Insulin human regular/ Insulin human isophane (NPH)  Insulin detemir  Insulin glargine  Insulin degludec |
| Metformin | Metformin |
| DDP4 inhibitors | Alogliptin, linagliptin, saxagliptin, sitagliptin |
| Sulfonylureas | Acetohexamide, chlorpropamide, tolazamide, tolbutamide, glipizide, glyburide, glimepiride |
| Meglitinide derivatives | Repaglinide, nateglinide |
| Alpha-glucosidase inhibitors | Acarbose, miglitol |
| Thiazolidinediones (TZDs) | Rosiglitazone, pioglitazone |
| Glucagonlike peptide–1 (GLP-1) agonists | Exenatide, liraglutide, lixisenatide, albiglutide, dulaglutide, semaglutide |
| SGLT2 inhibitors | Canagliflozin, dapagliflozin, empagliflozin, ertugliflozin |
| Pramlintide | Pramlintide |

**Supplementary table S2** **Sensitivity analysis** **for risk of diabetes treatment intensification and switching**

|  | **Patients** | **Events** | **Person-years** | **IR (95% CI)** | **HR_1_ (95% CI)** | **HR_2_ (95% CI)** |
| --- | --- | --- | --- | --- | --- | --- |
| **All intensification events (Insulin and non-insulin)** |  |  |  |  |  |  |
| Abatacept | 1,679 | 204 | 1,121.7 | 181.9 (158.5-208.6) | 1.0 (ref) | 1.0 (ref) |
| TNF inhibitors | 5,595 | 681 | 4244.5 | 160.4 (148.8-173.0) | 0.92 (0.78-1.07) | 0.90 (0.75-1.09) |
| Rituximab | 832 | 104 | 555.7 | 187.1 (154.4-226.8) | 1.02 (0.80-1.29) | 1.01 (0.80-1.29) |
| Tocilizumab | 690 | 74 | 455.1 | 162.6 (129.5-204.2) | 0.89 (0.69-1.17) | 0.90 (0.69-1.19) |
| Tofacitinib | 596 | 42 | 340.9 | 123.2 (91.1-166.7) | 0.65 (0.47-0.91) | 0.60 (0.43-0.85) |
| **Insulin intensification** |  |  |  |  |  |  |
| Abatacept | 1,709 | 88 | 1,212.7 | 72.6 (58.9-89.4) | 1.0 (ref) | 1.0 (ref) |
| TNF inhibitors | 5,726 | 258 | 4,672.7 | 55.2 (48.9-62.4) | 0.79 (0.62-1.00) | 0.79 (0.59-1.05) |
| Rituximab | 844 | 43 | 602.1 | 71.4 (53.0-96.3) | 0.97 (0.68-1.40) | 0.90 (0.62-1.30) |
| Tocilizumab | 702 | 37 | 480.4 | 77.0 (55.8-106.3) | 1.06 (0.72-1.56) | 1.12 (0.76-1.67) |
| Tofacitinib | 607 | 21 | 359.4 | 58.4 (38.1-89.6) | 0.78 (0.48-1.25) | 0.85 (0.52-1.39) |
| **Non-insulin intensification** |  |  |  |  |  |  |
| Abatacept | 1,693 | 116 | 1,183.6 | 98.0 (81.7-117.6) | 1.0 (ref) | 1.0 (ref) |
| TNF inhibitors | 5,655 | 425 | 4,459.1 | 95.3 (86.7-104.8) | 1.02 (0.83-1.25) | 1.00 (0.78-1.28) |
| Rituximab | 839 | 62 | 585.4 | 105.9 (82.6-135.9) | 1.07 (0.78-1.45) | 1.13 (0.83-1.54) |
| Tocilizumab | 697 | 38 | 480.3 | 79.1 (57.6-108.7) | 0.81 (0.56-1.17) | 0.79 (0.55-1.15) |
| Tofacitinib | 600 | 21 | 350.4 | 59.9 (39.1-91.9) | 0.58 (0.36-0.92) | 0.48 (0.30-0.77) |
| **Non-insulin switching** |  |  |  |  |  |  |
| Abatacept | 1,438 | 76 | 1,036.7 | 73.3 (58.6-91.8) | 1.0 (ref) | 1.0 (ref) |
| TNF inhibitors | 4,867 | 278 | 3,980.0 | 69.9 (62.1-78.6) | 0.99 (0.77-1.28) | 1.00 (0.74-1.34) |
| Rituximab | 664 | 43 | 470.7 | 91.4 (67.8-123.2) | 1.20 (0.83-1.75) | 1.17 (0.81-1.71) |
| Tocilizumab | 576 | 28 | 395.9 | 70.7 (48.8-102.4) | 0.96 (0.62-1.48) | 0.92 (0.59-1.43) |
| Tofacitinib | 512 | 21 | 302.8 | 69.4 (45.2-106.4) | 0.90 (0.56-1.46) | 0.93 (0.56-1.53) |
|  |  |  |  |  |  |  |

IR is per 1,000 person-years.
HR_1_ - Unadjusted Cox model
HR_2_ - Cox model adjusted for age, sex, index year, steroid use, renal failure, liver disease, number of previous biologic DMARDs, methotrexate use, hydroxychloroquine use, statin use, # oral antidiabetic drugs, # insulin drugs, # rheumatology visits, # primary care physician visits, # endocrinologist visits, Charlson comorbidity score, and type 1 diabetes at baseline
