# Supplementary material for: Erratum to: Diagnosis and treatment of patients with antiphospholipid syndrome: a mixed-method evaluation of care in The Netherlands
Source: Rheumatol Adv Pract. 2021 Jun 21;5(2):rkab028. doi: 10.1093/rap/rkab028 (PMC8908781; doi:10.1093/rap/rkab028)
Supplement: rkab028_Supplementary_Data [file rkab028_Supplementary_Data.pdf]

SUPPLEMENTARY MATERIAL

Interview guide

The interview guide was formulated by two rheumatologists and a clinical immunologist and tested by a sounding board consisting of patients and physicians of the ARCH APS working group. The ARCH APS working group exists of the following (former) members: Maarten Limper, Tammo Brunekreef, Julia Spierings, Gerard Jansen, Marc Bijl, Karina de Leeuw, Titia Lely, Renate van der Molen, Rolf Urbanus, Nyika Kruyt, Marcel van de Ree, Judith Potjewijd, Sander Otter, Gerie Brandts and Jamy Scheerhoorn-Pullen.

How many patients with APS are currently under your treatment?

What is your view on the following statements?
1. The care for patients with APS in The Netherlands is too fragmented.
2. The care for patients with APS would improve with the advent of expertise centres.
3. A shared care model (expertise centre – general hospital) is the preferred care model for APS.
4. The coordinating physician should discuss psychosocial support after diagnosis.
5. In order to improve communication between physicians from different centres, better support of communication is necessary.
6. The information provision to patients with APS is insufficient.
7. Every patient with APS should be discussed in multidisciplinary consultation.
8. Only medical specialists who see at least 10 patients with APS per year can treat them optimally.

Indicators for quality of care
<u>Which outcome indicators are most appropriate to evaluate the quality of care for APS?</u> Quality of life
Daily functioning
Measures of fatigue
Pregnancy outcome
Recurring thrombo-embolic events
Otherwise, namely;

<u>Which process indicators are most appropriate to evaluate the quality of care for APS?</u>
Patient satisfaction
Accessibility of care
Accessibility of information
% of patients that is diagnosed within 1 year after first symptoms
Structural interdisciplinary cooperation

Main challenges in APS care
<u>Which challenges affect the quality of care for APS to the strongest extent?</u>
The care for patients with APS in The Netherlands is too fragmented.
Information exchange between centres is insufficient.
Current information provision for patients does not meet the need.
Recognition of APS by general practitioners is insufficient.
Recognition of APS by medical specialists is insufficient.
Cooperation between rheumatologists and other disciplines within centres is insufficient.
Otherwise, namely;

Points for improvement

Which points contribute most to improving the quality of care for APS in terms of diagnosis and treatment?

Interactive guidelines for APS
Central database of all patients through which quality of care can be made visible and research can be coordinated
Possibility to log on to patient records from other hospitals (in case of shared care)
Creating agreements on multidisciplinary treatment
Increasing awareness among general practitioners
Offering specialized care for expertise centres on the local level
Centralization of care in at most 5 expertise centres
Otherwise, namely;

Which points contribute most to improving the quality of care for APS in terms of information provision?

Online accessible list of medical specialists with particular expertise
Accessible self-management programme for patients
Central point for online information provision, accessible to patients, general practitioners and medical specialists
Possibility for patients, general practitioners and medical specialists to ask questions to an expert
Developing e-learning for general practitioners, medical specialists and paramedical health personnel
Otherwise, namely:

Online survey

Questionnaire ARCH APS

Dear reader,
Thank you very much for your willingness to cooperate in this study to improve care for patients with the antiphospholipid syndrome (APS).
Through this questionnaire we hope to identify what is important to patients in the care for and treatment of this disease.
We are interested in your opinion! What do you consider to be truly important when it comes to quality of care? And how do you currently assess the quality of care and the quality of information provision regarding APS? Filling in this questionnaire is fully anonymous and takes approximately 15 minutes.
Thank you for your cooperation and time.

On behalf on the working group ARCH APS.

Questionnaire ARCH APS

1. <u>What is your age?</u>
    - …….. years

2. <u>Your sex?</u>
    - Male
    - Female

3. <u>Your marital status?</u>
    - Married or cohabiting?
    - Single

4. <u>Your level of education?</u>
    - Primary education
    - Secondary education
    - MBO
    - HBO
    - WO

5. <u>In what year was the diagnosis APS made?</u>
    - …… (year)

6. <u>How long before the diagnosis was made did you experience complaints?</u>
    - >5 years
    - 3-5 years
    - 2-3 years
    - 1 year
    - 6 months
    - <6 months
    - Do not know

7. <u>At what kind of hospital are you treated?</u> (Multiple options possible)
    - University hospital
    - General hospital
    - Not treated at a hospital

- Otherwise: …….
- Do not know

8. Do you have another rheumatological condition in addition to the antiphospholipid syndrome (APS)?
    - No (it is primary, so only APS)
    - SLE
    - Sjögren's syndrome
    - Dermatomyositis
    - Rheumatoid arthritis
    - Other rheumatological conditions: ……
    - Do not know

9. Which disease manifestations do you have? (Multiple options possible)
    - Deep venous thrombosis
    - CVA/brain infarct
    - TIA
    - Obstetrical manifestations (miscarriage, hypertensive disorders of pregnancy)
    - Thrombocytopaenia (low blood platelets)
    - Livedo reticularis (common skin finding with a net-like pattern)
    - Endocarditis (heart valve inflammation)
    - Migraine
    - Other: ….
    - Do not know

10. What treatment do you get? (Multiple options possible)
    - Sintrom, acenocoumarol
    - Marcoumar, fenprocoumoron
    - Ascal, acetylsalicylic acid, carbasalate calcium
    - Plavix, clopidogrel
    - Heparin injections (like fraxiparine or clexane)
    - Rivaroxaban, dabigatran, edoxaban, apixaban
    - Hydroxychloroquine, plaquenil
    - Prednisolon
    - Other
    - Do not know

11. Do you experience limitations in your daily functioning due to APS?
    - Yes
    - No

    If yes, can you give a grade from 0-1-2 (PGA score)

Few limitations		Many limitations
0	**1**	**2**

12. Do you experience limitations in (paid) work due to APS?
    - Yes
    - No

13. Do you experience limitations in travelling due to APS?

- Yes
- No

If yes, can you give a grade from 0-1-2 (PGA score)

**Few limitations**		**Many limitations**
**0**	**1**	**2**

14. Who is your coordinating physician?
    - Rheumatologist
    - Internist
    - Cardiologist
    - Neurologist
    - Other
    - Do not know

15. Are you also seen by other medical specialists? (Multiple options possible)
    - Rheumatologist
    - Internist
    - Cardiologist
    - Neurologist
    - Other
    - Do not know

16. What is your experience regarding cooperation of your coordinating physician with other medical specialists?
    - Very good
    - Good
    - Neutral
    - Poor
    - Very poor

17. How often are you seen per year (Multiple options available)
    - > 4x per year
    - 4x per year
    - 3x per year
    - 2x per year
    - 1x per year
    - Only in case of complaints
    - Other

18. Have you received sufficient information regarding APS when you received the diagnosis?
    - More than sufficient
    - Sufficient
    - Insufficient

19. If not, how could that be improved? (open question)

20. Were you offered psychological support when you heard about the diagnosis?
    - Yes
    - No, but no need for psychological support
    - No

21. Are you a member of the patient organization NVLE?
    - Yes
    - No

22. If not, do you know the patient organization?
    - Yes, but not yet a member
    - Yes, but no interest in becoming a member
    - No

23. Which outcome of care do you consider to be important? What should treatment for APS be focused on? (open question)

24. Do you miss certain things regarding the care for APS patients? (open question)

25. Do you have additional remarks that have not been addressed in this survey? (open question)

26. Would you like to receive more information about ARCH in the future?
    - No
    - Yes, I give permission to submit my email address to ARCH to receive more information in the future

    Your email address:

    ……………………………………………..

    Thank you for your cooperation!

Supplementary Table S1: Report Form (CRF) employed for medical record review

1.1	Year of birth	
1.2	Sex	Male<br>Female
1.3	Duration of disease in years	
1.3.1	*If 'Duration of disease in years' is smaller or equal than '1' answer this question*: Duration of disease in months	
1.4	Deceased	Yes<br>No
1.5	Time until first disease manifestation and under treatment of physician in months	
1.6	Time until under treatment of physician and diagnosis APS in months	
1.7	Primary/secondary APS	Primary APS<br>Secondary APS
1.7.1	*If 'Primary/secondary APS' is equal to 'Secondary APS' answer this question*: Secondary APS	SLE<br>RA<br>Sjögren's syndrome<br>Other
1.7.1.1	*If 'Secondary APS' is equal to 'Other' answer this question*: Other	
1.8	APS criteria up to now<br>(NB: 'weak positive' laboratory measurements are not considered to be positive.	Thrombosis<br>Pregnancy complications<br>LAC<br>aCL<br>anti-beta-2 GPI<br>Other<br>Unknown
1.8.1	*If 'APS criteria up to now' is equal to 'Thrombosis' answer this question*: Thrombosis	Arterial<br>Venous (excluding ocular)<br>Small vessel<br>Other<br>Unknown
1.8.1.1	*If 'Thrombosis' is equal to 'Other' answer this question*: Other thrombosis	
1.8.1.2	*If 'Thrombosis' is equal to 'Venous (excluding ocular)' answer this question*: Number of venous thrombosis	
1.8.1.3	*If 'Thrombosis' is equal to 'Arterial' answer this question*: Number of arterial thrombosis	
1.8.1.4	*If 'Thrombosis' is equal to 'Small vessel' answer this question*: Number of small vessel thrombosis	
1.8.2	*If 'APS criteria up to now' is equal to 'Pregnancy complications' answer this question*: Pregnancy complications	Miscarriage >10 weeks<br>Premature birth <34 weeks<br>Spontaneous abortion <10 weeks<br>Other<br>Unknown
1.8.2.1	*If 'Pregnancy complications' is equal to 'Miscarriage >10 weeks' answer this question*: Number of miscarriages	
1.8.2.2	*If 'Pregnancy complications' is equal to 'Premature birth <34 weeks' answer this question*: Number of premature births	
1.8.2.3	*If 'Pregnancy complications' is equal to 'Spontaneous abortion <10 weeks' answer this question*: Number of spontaneous abortions	
1.8.3	*If 'APS criteria up to now' is equal to 'LAC' answer this question*: Number of LAC	1

		2 or more
1.8.3.1	*If 'Number of LAC' is equal to '2 or more' answer this question*: 12 weeks between positive measurements?	Yes<br>No
1.8.4	*If 'APS criteria up to now' is equal to 'aCL' answer this question*: Number of aCL	1<br>2 or more
1.8.4.1	*If 'Number of aCL' is equal to '2 or more' answer this question*: 12 weeks between positive measurements?	Yes<br>No
1.8.5	*If 'APS criteria up to now' is equal to 'anti-beta-2 GPI' answer this question*: Number of anti-beta-2 GPI	1<br>2 or more
1.8.5.1	*If 'Number of anti-beta-2 GPI' is equal to '2 or more' answer this question*: 12 weeks between positive measurements?	Yes<br>No
1.8.6	*If 'APS criteria up to now' is equal to 'Other' answer this question*: Other criteria	
1.9	Non-criteria manifestations	Livedo reticularis<br>Migraine<br>Thrombocytopaenia<br>Insult<br>Cutaneous ulceration<br>Valvular heart disease<br>aPL-related nephropathy<br>Chorea<br>Superficial venous thrombosis<br>Other
1.9.1	*If 'Non-criteria manifestations' is equal to 'Other' answer this question:* Other non-criteria	
1.10	Treatment after diagnosis	Vitamin K-antagonist<br>Carbasalate calcium (ascal)<br>Acetylsalicylic acid<br>Clopidogrel<br>LMWH<br>DOAC/NOAC<br>HCQ<br>Other<br>Unknown<br>No treatment
1.10.1	*If 'Treatment after diagnosis' is equal to 'Other' answer this question:* Other treatment	
1.11	Events after diagnosis	Yes<br>No
1.11.1	*If 'Events after diagnosis is equal to 'Yes' answer this question:* Event after diagnosis	Thrombosis<br>Pregnancy complications<br>Other
1.11.2	*If 'Events after diagnosis is equal to 'Yes' answer this question:* Event under treatment	Yes<br>No<br>Unknown
1.11.2.1	*If 'Event under treatment' is equal to 'Yes' answer this question:* Which treatment	Vitamin K-antagonist<br>Carbasalate calcium (ascal)<br>Acetylsalicylic acid<br>Clopidogrel<br>LMWH<br>DOAC/NOAC<br>HCQ

		Other<br>Unknown<br>No treatment
1.12	Current treatment	Vitamin K-antagonist<br>Carbasalate calcium (ascal)<br>Acetylsalicylic acid<br>Clopidogrel<br>LMWH<br>DOAC/NOAC<br>HCQ<br>Other<br>Unknown<br>No treatment
1.2.1	*If 'Sex' is equal to 'Female' answer this question:* Other pregnancies	Yes<br>No<br>Not applicable<br>Unknown
1.2.1.1	*If 'Other pregnancies' is equal to 'Yes' answer this question:* Number of other pregnancies	
1.2.1.1.1	*If 'Other pregnancies' is bigger or equal than '1' answer this question:* Number of children born alive >37 weeks	
1.2.1.1.2	*If 'Other pregnancies' is bigger or equal than '1' answer this question:* Birth weight child 1	
1.2.1.1.3	*If 'Other pregnancies' is bigger or equal than '2' answer this question:* Birth weight child 2	
1.2.1.1.4	*If 'Other pregnancies' is bigger or equal than '3' answer this question:* Birth weight child 3	
1.2.1.1.5	*If 'Other pregnancies' is bigger or equal than '4' answer this question:* Birth weight child 4	
1.2.1.1.6	*If 'Other pregnancies' is bigger or equal than '5' answer this question:* Birth weight child 5	
1.13	Organ damage	Yes<br>No
1.13.1	*If 'Organ damage' is equal to 'Yes' answer this question:* Organ damage	Permanent ischemic events<br>Neurologic damage<br>Amputation<br>CAPS<br>Kidney failure<br>Heart failure<br>Other
1.13.1.1	*If 'Organ damage' is equal to 'Other' answer this question:* Other organ damage	
1.14	Involved specialists	Rheumatology/immunology<br>Haematology<br>Gynaecology<br>Neurology<br>Internal medicine<br>Cardiology<br>Other
1.14.1	*If 'Involved specialists' is equal to 'Other' answer this question:* Other specialists	
1.15	Clear coordinating physician APS	Yes<br>No
1.15.1	*If 'Clear coordinating physician APS' is equal to 'Yes' answer this question:* Which coordinating physician	Rheumatology/immunology<br>Haematology<br>Gynaecology<br>Neurology

		Internal medicine<br>Cardiology<br>Other
1.16	Possibility of multidisciplinary consultation	Yes<br>No<br>Unknown
1.16.1	*If 'Possibility of multidisciplinary consultation' is equal to 'Yes' answer this question:* Involved with multidisciplinary consultation	Rheumatology/immunology<br>Haematology<br>Gynaecology<br>Neurology<br>Internal medicine<br>Cardiology<br>Other
1.17	Number of hospitals where under treatment for APS	
1.18	Complications of treatment	Bleeding<br>Intolerance<br>Other<br>None
1.18.1	*If 'Complications of treatment' is equal to 'Other' answer this question:* Other complications	
1.19	Quality of life	Yes<br>No
1.19.1	Outcome quality of life	
1.20	Fitness for work	Fully fit for work<br>Not fit for work<br>Partly fit for work<br>Unknown
1.20.1	*If 'Fitness for work' is equal to 'Partly fit for work' answer this question:* Percentage fitness for work	
1.21	Involved specialized nurse/'reumaconsulent(e)'	Yes<br>No<br>Unknown
1.22.1	Other remarks	

Supplementary Table S2: COREQ (COnsolidated criteria for REporting Qualitative research) Checklist

Topic	Item No.	Guide Questions/Description	Reported
**Domain 1: Research team and reflexivity**			
Personal characteristics			
Interviewer/facilitator	1	Which author/s conducted the interview or focus group?	Setting and participants
Credentials	2	What were the researcher's credentials? E.g. PhD, MD	Setting and participants
Occupation	3	What was their occupation at the time of the study?	Setting and participants
Gender	4	Was the researcher male or female?	Setting and participants
Experience and training	5	What experience or training did the researcher have?	Setting and participants
Relationship with participants			
Relationship established	6	Was a relationship established prior to study commencement?	Setting and participants
Participant knowledge of the interviewer	7	What did the participants know about the researcher? e.g. personal goals, reasons for doing the research	Setting and participants
Interviewer characteristics	8	What characteristics were reported about the inter viewer/facilitator? e.g. Bias, assumptions, reasons and interests in the research topic	Setting and participants
**Domain 2: Study design**			
Theoretical framework			
Methodological orientation and Theory	9	What methodological orientation was stated to underpin the study? e.g. grounded theory, discourse analysis, ethnography, phenomenology, content analysis	Data analysis
Participant selection			
Sampling	10	How were participants selected? e.g. purposive, convenience, consecutive, snowball	Setting and participants
Method of approach	11	How were participants approached? e.g. face-to-face, telephone, mail, email	Setting and participants
Sample size	12	How many participants were in the study?	Results
Non-participation	13	How many people refused to participate or dropped out? Reasons?	Results
Setting			
Setting of data collection	14	Where was the data collected? e.g. home, clinic, workplace	Setting and participants
Presence of nonparticipants	15	Was anyone else present besides the participants and researchers?	Setting and participants
Description of sample	16	What are the important characteristics of the sample? e.g. demographic data, date	Table 1; Table 2; Supplementary data S4
Data collection			
Interview guide	17	Were questions, prompts, guides provided by the authors? Was it pilot tested?	Supplementary data S1

Repeat interviews	18	Were repeat inter views carried out? If yes, how many?	N/A
Audio/visual recording	19	Did the research use audio or visual recording to collect the data?	Data collection
Field notes	20	Were field notes made during and/or after the interview or focus group?	N/A
Duration	21	What was the duration of the inter views or focus group?	Setting and participants
Data saturation	22	Was data saturation discussed?	N/A
Transcripts returned	23	Were transcripts returned to participants for comment and/or correction?	N/A
**Domain 3: analysis and findings**			
Data analysis			
Number of data coders	24	How many data coders coded the data?	Data analysis
Description of the coding tree	25	Did authors provide a description of the coding tree?	N/A
Derivation of themes	26	Were themes identified in advance or derived from the data?	Data analysis
Software	27	What software, if applicable, was used to manage the data?	N/A
Participant checking	28	Did participants provide feedback on the findings?	N/A
Reporting			
Quotations presented	29	Were participant quotations presented to illustrate the themes/findings? Was each quotation identified? e.g. participant number	Supplementary data 5
Data and findings consistent	30	Was there consistency between the data presented and the findings?	Results, appendices
Clarity of major themes	31	Were major themes clearly presented in the findings?	Results
Clarity of minor themes	32	Is there a description of diverse cases or discussion of minor themes?	Results

Developed from: Tong A, Sainsbury P, Craig J. Consolidated criteria for reporting qualitative research (COREQ): a 32-item checklist for interviews and focus groups. *International Journal for Quality in Health Care*. 2007. Volume 19, Number 6: pp. 349 – 357

Supplementary Table S3: Responses of focus group participants, survey respondents and medical specialists

Demographic and clinical characteristics and experiences of focus group participants (n=14)

Age: mean (SD)		45.3	(11.7)
Sex: n (%)	Female	13	(92.9%)
	Male	1	(7.1%)
Marital status: n (%)	Married or cohabiting	9	(64.3%)
	Single	5	(35.7%)
Country of birth: n (%)	The Netherlands	14	(100%)
Highest completed education: n (%)	LBO, MAVO, VMBO (lower vocational training)	2	(14.3%)
	HAVO, VWO, MBO (middle vocational training and pre-university secondary education)	2	(14.3%)
	HBO (higher vocational training), university	10	(71.4%)
Duration of disease in years: mean (SD)		7.1	(6.0)
Current medication: n (%)	Acenocoumarol	8	(57.1%)
	Acetylsalicylic acid, carbasalate calcium	5	(35.7%)
	Hydroxychloroquine	3	(21.4%)
	Fenprocoumon	3	(21.4%)
	Prednis(ol)on	3	(21.4%)
	NOAC/DOAC	2	(14.3%)
	Heparin	2	(14.3%)
	Clopidogrel	1	(7.1%)
	Other	2	(14.3%)
Treatment centre: n (%)	University hospital	7	(50.0%)
	General hospital	5	(35.7%)
	Not under treatment at any hospital	2	(14.3%)
Duration of symptoms before diagnosis: n (%)	>5 years	3	(21.4%)
	3-5 years	0	(0%)
	2-3 years	3	(21.4%)
	1 year	0	(0%)
	6 months	1	(7.1%)
	<6 months	4	(28.6%)
	Do not know	3	(21.4%)
Other rheumatological disease: n (%)	No, primary APS	7	(50.0%)
	SLE	6	(42.8%)
	Sjögren's syndrome	1	(7.1%)
	Do not know	1	(7.1%)
Clinical manifestations	Deep venous thrombosis	3	(21.4%)
	Cerebrovascular accident	5	(35.7%)
	Transient ischemic attack	3	(21.4%)
	Obstetric manifestation	3	(21.4%)
	Thrombocytopenia	1	(7.1%)
	Livedo reticularis	5	(35.7%)
	Endocarditis	1	(7.1%)
	Migraine	6	(42.8%)
	Other	7	(50.0%)
Experienced limitation in daily functioning due to APS: n (%)	Yes	6	(42.8%)
	No	4	(28.6%)
	Do not know	4	(28.6%)
Experienced limitation in work due to APS: n (%)	Yes	6	(42.8%)
	No	7	(50.0%)
	Do not know	1	(7.1%)
Experienced limitation in travel due to APS: n (%)	Yes	8	(57.1%)
	No	5	(35.7%)
	Do not know	1	(7.1%)
Coordinating physician(s): n (%)	Internist	7	(50.0%)
	Rheumatologist	5	(35.7%)
	Cardiologist	1	(7.1%)
	Neurologist	0	(0%)
	Other/do not know	3	(21.4%)
Number of hospital visits per year: n (%)	>4	5	(35.7%)
	4	2	(14.3%)
	3	1	(7.1%)

	2	0	(0%)
	1	4	(28.6%)
	Other/do not know	2	(14.3%)
Information provision after diagnosis: n (%)	Abundant	0	(0%)
	Sufficient	3	(21.4%)
	Insufficient	11	(78.6%)
Offered psychosocial support after diagnosis: n (%)	Yes	4	(28.6%)
	No, despite need for psychosocial support	6	(42.8%)
	No, no need for psychosocial support	4	(28.6%)
Member of patient organization (NVLE): n (%)	Yes	9	(64.3%)
	No, but familiar with patient organization	5	(35.7%)
	No, not familiar with patient organization	0	(0%)

Demographic and clinical characteristics and experiences of questionnaire respondents (n=79)

Age: mean (SD)		53	(11.3)
Sex: n (%)	Female	68	(86.1%)
	Male	11	(13.9%)
Marital status: n (%)	Married or cohabiting	64	(81.0%)
	Single	15	(19.0%)
Highest completed education: n (%)	Primary education	1	(1.2%)
	Secondary education	17	(21.5%)
	MBO (middle vocational training)	27	(34.15)
	HBO (higher vocational training)	23	(29.1%)
	University	11	(13.9%)
Duration of disease in years: mean (SD)		11	(8.9)
Current medication: n (%)	Acenocoumarol	27	(34.2%)
	Acetylsalicylic acid, carbasalate calcium	21	(26.6%)
	Hydroxychloroquine	24	(30.4%)
	Fenprocoumon	22	(27.8%)
	Prednis(ol)on	9	(11.4%)
	NOAC/DOAC	0	(0%)
	Heparin	3	(3.7%)
	Clopidogrel	5	(6.3%)
	Other	19	(24.1%)
Treatment centre: n (%)	University hospital	67	(84.8%)
	General hospital	6	(7.6%)
	Both university and general hospital	2	(2.5%)
	No treatment	3	(3.7%)
	Do not know	1	(1.2%)
Duration of symptoms before diagnosis: n (%)	>5 years	29	(36.7%)
	3-5 years	4	(5.1%)
	2-3 years	3	(3.8%)
	1 year	5	(6.3%)
	6 months	8	(10.1%)
	<6 months	11	(13.9%)
	Do not know	19	(24.1%)
Other rheumatological disease: n (%)	No, primary APS	33	(41.7%)
	SLE	21	(26.6%)
	Sjögren’s syndrome	4	(5.1%)
	Rheumatoid arthritis	3	(3.7%)
	Other (amongst others fibromyalgia, arthrosis, Raynaud)	14	(17.7%)
	Do not know	10	(12.6%)
Clinical manifestations	Deep venous thrombosis	37	(46.8%)
	Cerebrovascular accident	23	(29.1%)
	Transient ischemic attack	18	(22.7%)
	Obstetric manifestation	30	(38.0%)
	Thrombocytopenia	11	(13.9%)
	Livedo reticularis	10	(12.7%)
	Endocarditis	5	(6.3%)
	Migraine	12	(15.2%)
	Other	18	(22.8%)
Experienced limitation in daily functioning due to APS: n (%)	Yes	43	(54.4%)
	No	34	(45.6%)

Experienced limitation in work due to APS: n (%)	Yes	40	(50.6%)
	No	39	(49.4%)
Experienced limitation in travel due to APS: n (%)	Yes	34	(43.0%)
	No	45	(57.0%)
Coordinating physician(s): n (%)	Internist	18	(22.7%)
	Rheumatologist	36	(45.6%)
	Cardiologist	1	(1.3%)
	Neurologist	2	(2.5%)
	Immunologist	8	(10.1%)
	Other/do not know	14	(17.7%)
Experienced cooperation between physicians: n (%)	Very good	23	(29.1%)
	Good	27	(34.2%)
	Neutral	9	(11.4%)
	Poor	8	(10.1%)
	Very poor	1	(1.3%)
	Do not know	11	(13.9%)
Number of hospital visits per year: n (%)	>4	11	(13.9%)
	4	7	(8.9%)
	3	12	(15.2%)
	2	16	(20.3%)
	1	26	(32.9%)
	Other/do not know	14	(17.7%)
Information provision after diagnosis: n (%)	Abundant	11	(13.9%)
	Sufficient	29	(36.7%)
	Insufficient	33	(41.8%)
	Do not know	6	(7.6%)
Offered psychosocial support after diagnosis: n (%)	Yes	7	(8.9%)
	Yes, but no need for psychosocial support	6	(7.6%)
	No, despite need for psychosocial support	46	(58.2%)
	No, no need for psychosocial support	20	(25.3%)
Member of patient organization (NVLE): n (%)	Yes	24	(30.4%)
	No, but familiar with patient organization	25	(31.6%)
	No, not familiar with patient organization	30	(38.0%)

Relevant indicators of quality of APS care as reported by medical specialists and patients

**Relevant indicators of quality of APS care as reported by medical specialists (n=14)**			
Outcome measures of quality of care: n (%)	Thrombo-embolic events	12	(85.7%)
	Pregnancy outcomes	12	(85.7%)
	Quality of life	4	(28.6%)
	Bleeding complications	4	(28.6%)
Process measures of quality of care: n (%)	Presence of multidisciplinary consultation	8	(57.1%)
	Guideline adherence	6	(42.9%)
	Patient satisfaction	5	(35.7%)
	Time until diagnosis and/or treatment	3	(21.4%)
	Accessibility of care and information	2	(14.2%)
	Presence of specialized nurse	1	(7.14%)
	Specialist satisfaction	1	(7.14%)
	Involvement of expertise centre	1	(7.14%)
Main challenges in APS care: n (%)	Insufficient recognition by medical specialists	10	(71.4%)
	Insufficient recognition by general practitioners	7	(50.0%)
	Fragmentation of care	6	(42.9%)
	Insufficient information exchange between centres	4	(28.6%)
	Absence of clear, evidence-based guidelines	3	(21.4%)
	Insufficient provision of information to patients	3	(21.4%)
	Insufficient cooperation between specialists within centres	3	(21.4%)
	Variance in treatment	1	(7.14%)
	Inability to localize expertise	1	(7.14%)
	Quality of laboratory diagnostics	1	(7.14%)
Areas for improvement in APS care: n (%)	Shared care	6	(42.9%)
	Increasing awareness among general practitioners	5	(35.7%)
	Central database of all patients	4	(28.6%)
	Creating agreements on multidisciplinary treatment	4	(28.6%)
	Interactive guidelines	3	(21.4%)

	Improved electronic communication	3	(21.4%)
	Increasing awareness among medical specialists	3	(21.4%)
	Centralization of care in expertise centres	2	(14.3%)
	Visibility of expertise centres	1	(7.14%)
	Guideline for peri-operative management	1	(7.14%)
	Reliable information for patients	1	(7.14%)
	Harmonization of work	1	(7.14%)
	Scientific research	1	(7.14%)
	Guideline for laboratory diagnostics	1	(7.14%)
**Relevant indicators of quality of APS care as reported by survey respondents (n=79)**			
Outcome and process measures of quality of care: n (%)	Information provision	21	(26.6%)
	Disease manifestations	17	(21.5%)
	Quality of life	14	(17.7%)
	Feeling heard by coordinating physicians	11	(13.9%)
	Information exchange between physicians	6	(7.6%)
	Regular and easy contact with coordinating physician	6	(7.6%)
	Follow-up, including psychosocial support, care at home, occupational physician, etc.	6	(7.6%)
	Diagnostic process	5	(6.3%)
Main challenges and areas for improvement in APS care: n (%)	Information provision	17	(21.5%)
	Clarity	14	(17.7%)
	Awareness of APS among physicians	8	(10.1%)
	Follow-up, including psychosocial support, care at home, occupational physician, etc.	8	(10.1%)
	Experienced recognition of complaints by coordinating physician	7	(8.9%)
	Delayed diagnosis	6	(7.6%)
	Possibility to easily ask questions regarding APS	4	(5.1%)
	Peer support for patients and/or partners	2	(2.5%)
	Improved treatment	1	(1.3%)
	Document or wallet-sized card describing APS	1	(1.3%)
	Peri-operative management	1	(1.3%)

Translated illustrative responses of patients and medical specialists

**Diagnostic process**	Specialists	*"Maybe you would not even need patients visiting multiple hospitals, but when you have a patient and you need more expertise, knowing who to call or who can advise you." (1)*<br><br>*"I think that general practitioners tend to refer, especially when someone has a deep vein thrombosis for the second time. (…) So, I do not think that there is a long delay at the general practitioners." (3)*<br><br>*"Having a national diagnostic protocol for APS, agreed upon and upheld by all specialisms, would really help. Still too often you hear and notice that people treat the diagnostic process differently. […] This automatically brings people together in thinking about the current state of knowledge and the gaps. Now some authorities confidently say things that have not been sufficiently proven yet. These kinds of things create chaos, also amongst patients." (11)*
	Patients	*"I really felt tossed aside by the general practitioner. She literally said: 'Yes, that's stress. It's nothing, just a headache.' The circuit you end up in is really frustrating. I had complaints and as I do not consider myself someone to overreact, they had to come from somewhere. They only really reacted after my epileptic insult in 2009. Then they realized the complaints had an underlying cause. Initially they send me home from the emergency care, but at home I had three heavy epileptic insults and finally all the alarm bells went off. In hindsight I found this. very frustrating; that it had to come this far before they undertook any action. […] Yes now, in retrospect, he acknowledged that he hadn't been alert enough and apologized to me. I really appreciated that." (4)*<br><br>*"In my opinion, (name physician) deserves a statue. She diagnosed me, sent me to the [university hospital], and told me: 'When you have been there, please come back and tell me what they did.' Well, I thought that was golden, honestly. I believe that is really special." (5)'*<br><br>*"In hindsight, I would have wanted to go to an university hospital where there is the right knowledge and expertise. At first, my general practitioner took me seriously, but they let it go, a kind of pride: we can handle it here. No one thinks: I feel insecure about this, this is the limit of my knowledge, I refer you." (7)*<br><br>*"That was one of the most important things; really being heard. If I would say something that is completely disregarded, I wouldn't feel taken seriously anymore. That seems to me to be such a bleak*

		feeling. But I did not encounter that at all. They also clearly tell me that my insecurities should be taken away and I consider that really important as well." (10)<br><br>*"Acknowledgement, that's what I was just talking about. Not just recognition, but also affirmation. Sometimes I'm glad that I have had a heart attack and a stroke, because whenever I have something, they immediately take me seriously. That they don't send you away with complaints." (11)*<br><br>*"I had a similar thing: pulmonary embolisms and SLE. One plus one makes two, that is APS, I thought. They were incredibly surprised that I even knew the word APS, but it did really speed things up. And otherwise I would have been sent home, diagnosed with a double pneumonia, with antibiotics, and I would have just fiddled away. I was completely out of breath, couldn't climb stairs. I could do practically nothing, but I just thought: 'I don't have any stamina, my SLE is bad.' That is a pitfall."*<br>*"That was the same for me obviously. Since I suggested APS myself, they immediately pushed through and tested me for it. That was positive, even though I came up with it myself. However, if you enter with a fever of 40 degrees, you usually don't take the lead." (13)*
**Management after diagnosis**	Specialists	*"All these patients are being followed up at two or three policlinics without clear interaction between them. The communication and coordination between physicians, that is something that can be organized in a multidisciplinary consultation." (5)*<br><br>*"Yes, I regard our knowledge as a whole as too flimsy, the lack of evidence. With great confidence therapies are started that will never be stopped. This impedes the gathering of evidence and I consider this an enormous problem. Not just in the Netherlands. You read several reviews of big names in a country without evidence-based therapies that tell you to always do this or that. I even have a presentation on this. There is just no evidence regarding disease management during pregnancy. We know nothing. I find that really difficult." (7)*<br><br>*"It is a major problem that a patient can go shopping because the neighbouring hospital gives you a different treatment. That also leads to tremendous insecurity among patients." (8)*<br><br>*"When you need other specialists, they do not know the disease well enough. It is the multidisciplinary part that is not always working out well and which takes a lot of effort to explain to other specialists." (10)*
	Patients	*"I was visiting the cardiologist and he was like: 'Well sir, the heart looks good, so goodbye.' To which I replied: 'Goodbye? I learned that antiphospholipid antibodies mostly work on the valves and such, don't I need an ultrasound?' 'Oh right, that might be a good idea!' You know, I'm telling the cardiologist what to do. Then I really think that it would be better to have a multidisciplinary centre where you go one a year to get a full check-up. (….) I needed an open-heart surgery and the protocols were completely unclear, but I know that I cannot stop with my anticoagulant medication. Right before the surgery you get a call from the hospital: 'Yes, you are planned to have surgery the day after tomorrow, so you can already stop with the anticoagulants.' 'Well I don't think so. Seems to me that it should go differently.' 'No no, we called the doctor and he says you should stop.' 'Well, I'll only stop if my rheumatologist tells me to.' And then after a few curves and some steps sideways the rheumatologist comes and says that stopping indeed would not be a good idea." (1)*<br><br>*"With angst I await the moment that I'm unconscious or somehow unable to interfere. Then I will be in the hands of the person in charge at the moment and dependant on the knowledge of that person." (2)*<br><br>*"As a patient I always check my electronic files and really keep track of them. However, it shouldn't be the responsibility of the patient, but the information exchange between practitioners should just work. … As a patient you have to keep repeating that you use anticoagulants. Whenever someone suggests giving me ibuprofen, then I tell them that I can't have that because of my anticoagulant medication. (…) Despite it being in your file, they still prescribe you ibuprofen. (…) I would appreciate it if there would be an anticoagulation service especially for rare diseases where people work that are up to date in terms of knowledge and new developments. People who don't just think: Oh, you need a new dosage. […] It's just another situation if you have a rare disease that's dependant on so many factors. (3)*<br><br>*"I arranged with my physician in [university hospital] that he makes prints for my physician in [general hospital]. So, I can take a letter with me to discuss further actions, especially since it has gone wrong in the past. He just writes his reports on the computer and prints them so that I can take them with me." (4)*<br><br>*"I go to an eye doctor in a different hospital and he once said: 'I assume that the internist does the blood tests.' So, I said: 'You should really tell him that.' To which he responded: 'Oh, he doesn't work in this hospital?'" (6)*<br><br>*"I know that I can send messages to my internist via my electronic health record if I have questions. Even on his holiday, he replied to my questions! I was really happy about that." (8)*

		"I got my diagnose quite recently, so it is not all clear yet. Personally, I feel like a ping-pong ball." (9)
**Information provision, psychosocial support and functioning**	Specialists	*"Just like with any other disease, as a physician you have to be aware of the impact a new diagnosis has on a live and how differently people experience it. You should talk about this with your patient if you think that the impact is too much to cope with or when the patient needs help processing. Of course, you have to consider psychosocial support."(6)*<br><br>*"It is very disappointing. For physicians there is little scientific information available, for patients there is even less. It's only mouth-to-mouth and very poor." (9)*<br><br>*"I'm not sure whether it helps if patients look for information themselves, even though it's a good source. Still then it is hard to distil what applies to them, to their specific situation." (13)*<br><br>*"A lot of patients have had a deep vein thrombosis once and another one later. Heaps of people had this in the Netherlands without ever finding the cause. Those people are put on anticoagulation without guidance. If by coincidence primary APS is found without cardiac or neurological complications or wish to become pregnant, those people are almost like you and me. They don't need psychosocial support." (14)*
	Patients	*"Personally, I would really like to read scientific articles, but those are often untraceable for patients. (…) That knowledge should be available for patients, because you have to know everything very well since you're seeing so many different doctors. (…) So, I am self-employed and thus don't have a safety net. […] I work fulltime. But if I have to stop working before my pregnancy leave starts due to complications, I lose income, there's no safety net." (3)*<br><br>*"And he (the cardiologist) had already answered to four of my questions: "Well mam, I can tell you yes and no, but the honest answer is that I have no clue. 'My dear', I thought, 'what on earth is wrong with me? I'm in an university hospital sitting down with someone with two titles in front of his name, what is going on?' That made me really anxious. (5)*<br><br>*"Last summer my general practitioner called me after I had a pulmonary embolism because he still received so many letters of referral. He just wanted to know how I was doing. I was so surprised, but it was so nice. Those kinds of things really help." (8)*<br><br>*"And I still don't know what that antibody means. Is that APS? I don't know. And I'm not getting from my gp, my rheumatologist, my internist, nobody gives me uh – I either have to go get a scan or take photos of my lungs, a cardiogram, all those kinds of things, but clarity about anything, I won't get that." "I have everything from that one site, the NVLE [Dutch patient organization], everything I know. And I Google a lot myself, but you encounter so many horror stories as well, so many." (12)*<br><br>*"I was really worried, really anxious even because I know a lot of people from the patient organization with SLE and APS. Most have had heart attacks, strokes, you name it. A lot of scary things. I worried that that was awaiting me. So, I was not only terribly ill, but also terrified. However, no one ever asked me whether I wanted psychological help. I would have really appreciated it if someone could have reassured me. In hindsight I realize that no one could have put me at ease. There is no clarity. I understand that now, but at the moment I really couldn't. I was so afraid. (…) You don't trust your own body anymore and you don't trust the doctor either. You're in no-man's-land." (13)*<br><br>*"I have APS now and I think there's a chance that it will turn into SLE as well. Or what if I get an epileptic insult? I don't know who will pick me up, so I find that real scary. What is awaiting me? (…) Sometimes I'm forgetful and everyone tells me that that happens to them as well. I'm sure that that's the case and that it comes with aging, but I really would like to know whether it's not more than just my age." (14)*
